# Electrospun Co Nanoparticles@PVDF-HFP Nanofibers as Efficient Catalyst for Dehydrogenation of Sodium Borohydride

**DOI:** 10.3390/polym15030597

**Published:** 2023-01-24

**Authors:** Ahmed Abutaleb, Ibrahim M. Maafa, Nasser Zouli, Ayman Yousef, M. M. El-Halwany

**Affiliations:** 1 Department of Chemical Engineering, College of Engineering, Jazan University, Jazan 45142, Saudi Arabia; 2 Department of Mathematics and Physics Engineering, College of Engineering in Matteria, Helwan University, Cairo 11718, Egypt; 3 Department of Mathematics and Physics Engineering, College of Engineering, Mansoura University, El-Mansoura 35516, Egypt

**Keywords:** PVFH NFs, cobalt, hydrolysis, hydrogen, sodium borohydride, electrospinning

## Abstract

Metallic Co NPs@poly(vinylidene fluoride-co- hexafluoropropylene) nanofibers (PVFH NFs) were successfully synthesized with the help of electrospinning and in situ reduction of Co^2+^ ions onto the surface of PVFH membrane. Synthesis of PVFH NFs containing 10, 20, 30, and 40 wt% of cobalt acetate tetrahydrate was achieved. Physiochemical techniques were used to confirm the formation of metallic Co@PVFH NFs. High catalytic activity of Co@PVFH NFs in the dehydrogenation sodium borohydride (SBH) was demonstrated. The formulation with 40 wt% Co proved to have the greatest performance in comparison to the others. Using 1 mmol of SBH and 100 mg of Co@PVFH NFs, 110 mL of H_2_ was produced in 19 min at a temperature of 25 °C, but only 56, 73, and 89 mL were produced using 10, 20, and 30 wt% Co, respectively. With the rise of catalyst concentration and reaction temperature, the amount of hydrogen generated increased. By raising the temperature from 25 to 55 °C, the activation energy was lowered to be 35.21 kJ mol^−1^ and the yield of H_2_ generation was raised to 100% in only 6 min. The kinetic study demonstrated that the reaction was pseudo-first order in terms of the amount of catalyst utilized and pseudo-zero order in terms of the SBH concentration. In addition, after six cycles of hydrolysis, the catalyst showed outstanding stability. The suggested catalyst has potential applications in H_2_ generation through hydrolysis of sodium borohydride due to its high catalytic activity and flexibility of recycling.

## 1. Introduction

Hydrogen (H_2_) has a high calorific value and has been highlighted as a long-term renewable, clean, environmentally friendly energy carrier for future use that can meet the increasing need for clean energy throughout the world [1]. Over the past decade, researchers have explored many methods of practically storing hydrogen, with the most promising being the storage of hydrogen as a chemical hydride [2,3]. With its high H_2_ capacity [4,5], outstanding stability in alkaline solution [5,6], recycling of by-products [6,7,8], non-flammability, and cheap cost, sodium borohydride (SBH, NaBH_4_) has garnered a lot of interest as a desirable material for H_2_ storage and generation [9]. Theoretically, one mole of SBH at room temperature may break down into four moles of H_2_ in water with a suitable catalyst present, with four equivalent H atoms originating from the SBH itself and the remaining two moles coming from the breakdown of H_2_O, as shown in Equation (1).

(1) $$NaBH_{4}+2H_{2}O\overset{catalyst}{\to }4H_{2}+NaBO_{2}$$

Because self-hydrolysis of SBH hydrolysis is sluggish, adding a suitable catalyst allows for continuous-flow H_2_ from SBH hydrolysis under mild conditions, which is ideal for practical applications [10]. The hydrolysis process of NaBH_4_ has previously been accomplished using a wide variety of nano-catalytic materials [4,11,12,13,14,15,16,17,18,19], including Pt, Ru, Rh, Pd, Ni, Co, Fe, and their alloys. Co has emerged as the most viable option because of its strong catalytic activity and improved durability [20,21]. Its reactivity is comparable to that of noble metals, yet it is significantly cheaper [22,23]. Common methods for producing metal nano-catalysts include reducing metal salts with reductants like NaBH_4_ or hydrazine hydrate. The following practical difficulties affect metallic particles:

1. The catalysts used are losing activity and cycle stability [24];
2. The Co catalyst becomes ineffective quickly because a layer of B-O compounds is deposited on top of it to function as a passivation layer;
3. Powdered metal-based catalysts are widely used, however this form of the catalyst is difficult to use in start-and-stop applications because the powder may easily be separated from the reaction fluid [25];
4. They tend to cluster together frequently.

Encapsulation of Co NPs in an appropriate substrate is one method suggested to lessen Co aggregation and leaching. Dispersing and stabilizing cobalt metal particles over a suitable substrate enhances the catalytic activity of cobalt metal-based catalysts [15]. It is well known that the support enhances the overall surface area of the catalyst and facilitates more even distribution of the active metal particles. Different appropriate supporting materials are proposed during the preparation procedure (e.g., SiO_2_ [26], Al_2_O_3_ [27], TiO_2_ [28,29], CeO_2_ [30], carbon materials [3,31,32], polymer materials [1,2,11,24,33,34], etc.) and can help maintain the catalyst’s structural stability, achieve long-term durability, and improve catalytic activity while also addressing the aforementioned concerns [35,36]. As is well-known, polymer substrates may easily be isolated from reactants while yet retaining their flexible design structures. For H_2_ production from SBH hydrolysis, Lunhong et al. [24] produced metallic cobalt@macroscopic alginate hydrogels as an efficient and reusable catalyst. The catalytic activity and stability of catalysts are, as mentioned before, strongly influenced by their synthesis method and shape. In a number of chemical processes [37,38,39], polymer membrane nanofibers (PMNFs) have been employed as a substrate for other NPs. They can be broken down into their component parts and recycled effortlessly, and they perform well. Since NFs may interlace to produce a very porous mesh, their potential applications are almost unlimited [9]. Li et al. [40] as a result of anchoring CoCl_2_ on PAN NFs were able to produce H_2_ from SBH solution with high catalytic performance and stability. Using polyvinylidene fluoride (PVF) as a support matrix, Kim and colleagues have produced hybrid NFs for SBH dehydrogenation, such as CoCl_2_-PVF [9], and Y-zeolite/CoCl_2_-PVF. They have demonstrated great catalytic performance and flexible reusability. However, the polymer’s hydrophobicity dampened its catalytic potential. In order to generate H_2_ from SBH, Yingbo et al. [41] utilized PVDF-Ni hollow fiber membranes prepared with hydrophilic polyethylene glycol. To better distribute the catalyst and decrease the hydrophobicity of PVF, polyethylene glycol was used in the catalyst layer production process. Electrospun PVFH NFs have been developed as a polymer electrolyte and membrane in diverse applications in recent years [42,43,44,45,46]. They have excellent chemical and electrochemical stability and a high affinity for absorbing electrolyte solution [47]. The tetrahydrate content of the precursor used in this work means that the deposited NPs are superior to those that are embedded inside the PVFH NFs, therefore the hydrophobic surface of the PVFH NFs was put to good use. The polymer’s crystallinity is lowered by this hypothesis, and the solution absorption is boosted, which may lead to better SBH–catalyst surface contact [42]. Since PVFH NFs are an unreactive and simply reusable polymer, they are a promising candidate for use as NPs’ supporting material. The fabrication of Co NPs@PVFH NFs’ membrane NFs for dehydrogenation of SBH as a reliable and simply recyclable catalytic hybrid NF has not been previously studied, to the best of our knowledge. In this research, economical catalysts for the dehydrogenation of SBH were explored, namely metallic Co nanoparticles supported on PVFH NFs. Electrospinning and a chemical reduction method were used to produce the introduced NFs. CoAc and PVFH NFs electrospun nanofibers were dried and afterwards reduced in situ with SBH in methanol medium to yield Co NPs supported on PVFH NFs’ NFs. According to the physicochemical characterization techniques used, PVFH NFs’ NFs with Co supported on them are formed during the reduction process. H_2_ was successfully produced from SBH using the proposed NFs, demonstrating their superior catalytic activity. The superb dispersion and stability of the Co nanoparticles in the Co@PVFH NFs catalyst gives them remarkable catalytic activity and a remarkable recycling property.

## 2. Experimental

### 2.1. Materials

The following chemicals were obtained from Aldrich Co., St. Louis, MO, USA: cobalt (II) acetate tetrahydrate (CoAc, 98% assay), PVFH (98% assay, 65,000 g/mol), SBH (98% assay), N,N, dimethylformamide (DMF, reagent grade, 99% assay), and acetone.

### 2.2. Hybrid Membrane Preparation

To begin, a solution containing 15.0 wt% PVFH was formed by dissolving 1.5 gm of PVDF-HFB in a solution including DMF and acetone with a weight ratio of 4:1. The cobalt-supported PVDF-HFB membrane was made in four different formulations, each of which had a different amount of CoAc loading (10.0 (PVFH-10), 20.0 (PVFH-20), 30.0 (PVFH-30), and 40.0 wt% (PVFH-40). In order to get the desired concentration of CoAc, it was first soluble in DMF and afterwards mixed with the PVFH solution. The solutions were agitated for 5 h in a stirrer to ensure a uniform sol-gel. Lab-scale electro-spinner equipment was utilized to spin the prepared sol-gels. Following its preparation, the sol-gel was transferred to a syringe. A high-voltage power source was connected to one end of the syringe, and a copper wire was inserted through the syringe (positive electrode). Attached to the negative electrode of a high-voltage power source was a ground iron drum covered in aluminum foil. It was subjected to a 20 kV electric field. In order to dry the collected formed membranes, they were placed in a vacuum dryer at 40 °C for one whole day. In order to prepare a pure PVFH-free CoAc membrane, the identical technique was carried out.

### 2.3. In Situ Reduction of Co Ions Supported on PVFH Membranes

A predetermined amount of electrospun NFs mat was soaked in a 500 mL beaker that contained a defined amount of SBH in methanol solution. This method boosts the Co ions’ reduction more than the water medium does. In order to achieve a full reduction process, the molar ratios of the metal precursor and the SBH were set as 1:5. Immediately after the attachment of the membranes to the solution, the membrane NFs changed color, going from purple to black. The membranes continued to be placed on to the solution until the gas bubble was no longer visible. The black membrane NFs was rinsed many times with DI water to remove any residues that may have been left behind. In the end, the membrane NFs were heated to 30 °C overnight while being dried in a vacuum.

### 2.4. Characterization

The Co@PVFH membranes were characterized in a similar fashion to that in our recent reports [48].

### 2.5. Dehydrogenation of SBH Used Co@PVFH Membranes

Round-bottomed Pyrex flasks were utilized for the catalytic reaction. The water displacement technique was used to determine the volume of H_2_ generated. The reaction vessel was kept in a water bath with a thermostat in order to keep the temperature constant. The proper amount of catalysts and aqueous SBH solution were introduced to the reaction vessel. Using the water displacement method, hydrogen gas was produced during the reaction and collected in an upside-down burette. Hydrogen production was determined by monitoring the rise and fall of water levels in burettes at regular intervals. The kinetics of the hydrolysis process were studied by varying the catalyst amount, the SBH concentration, and the temperature. The lifetime of the newly added Co@PVFH membranes via recycling was also investigated.

## 3. Results and Discussion

**Figure 1:**
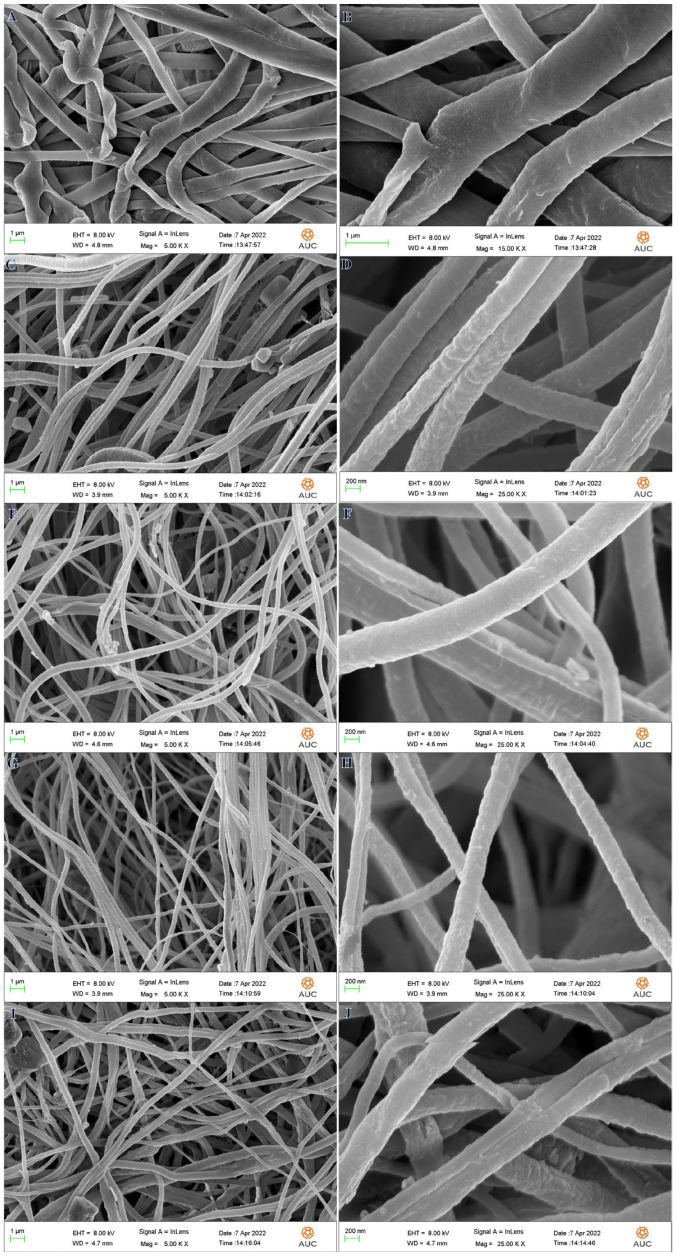
SEM pictures of PVFH (**A**,**B**), PVFH-10 (**C**,**D**), PVFH-20 (**E**,**F**), PVFH-30 (**G**,**H**), and PVFH-40 (**I**,**J**) membranes.

Increased interconnectivity, flexibility, perfect porosity, and remarkable surface-to-volume ratios are just a few of the potential benefits of a polymeric nanofibrous membrane produced by the electrospinning method [49,50]. Polyvinylidene fluoride (PVDF-HFP) is the polymeric material of preference for making these NF sheets [51,52] because of its semi-crystalline structure, high thermal stability, enhanced dielectric constant, and hydrophobicity, in addition to its piezo and pyroelectric properties. Dry electrospun PVFH membranes are exhibited in low and high magnification SEM images in Figure 1A,B, respectively, demonstrating an excellent nanofibrous structure. Because the acetone solvent quickly evaporates during electrospinning, before the NFs reach the surface of the drum, the nanoporous structure is formed, which is beneficial for the nucleation of Co crystals. The cobalt precursor salt used in this study contained a high water content, which greatly increased the hydrophilicity of the synthesized polymeric membranes by increasing the demixing rate within the liquid–liquid phases, thereby favoring the formation of a dense network of pores on the membrane’s structure [53]. Once there, the analyte molecules would have the least barrier to diffusion, and would be readily trapped in the pores to make way for the development of H_2_. Incorporating metal salts has a favorable effect on improving conductivity and gel formation of the polymer solution, which in turn leads the jet to stretch to its utmost extent along its axis and forms polymeric nanofibers of very small diameters [54]. Metallic Co NPs are formed and coated the PVHP membrane surface by in situ reduction of Co ions using SBH as a strong and effective reducing agent in a MeOH medium. In Figure 1C,D (PVFH-10), E,F (PVFH-20), G,H (PVFH-30), and I,J (PVFH-40), SEM images of Co@PVFH membranes are exhibited at low and high magnifications. The formation of rough, bead-free NFs is seen in the figure. In addition, PVFH mats are coated in decreased Co ions because they are developed on the basis of the nano-pores existing on the PVFH NFs’ surface.

The by-product of methanolysis is sodium tetra methoxy borate (NaB (OCH_3_)_4_, which may be expressed as follows in Equation (2). Sodium tetra methoxy borate has a chemical reaction with water (Equation (3)), yielding sodium borate and methanol, both of which are removed from the system by the washing process.

(2) $$NaBH_{4}+4CH_{3}OH\to 4H_{2}+NaB{\left(OCH_{3}\right)}_{4}$$

(3) $$NaB{\left(OCH_{3}\right)}_{4}+2H_{2}O\to NaBO_{2}+4CH_{3}OH$$

**Figure 2:**
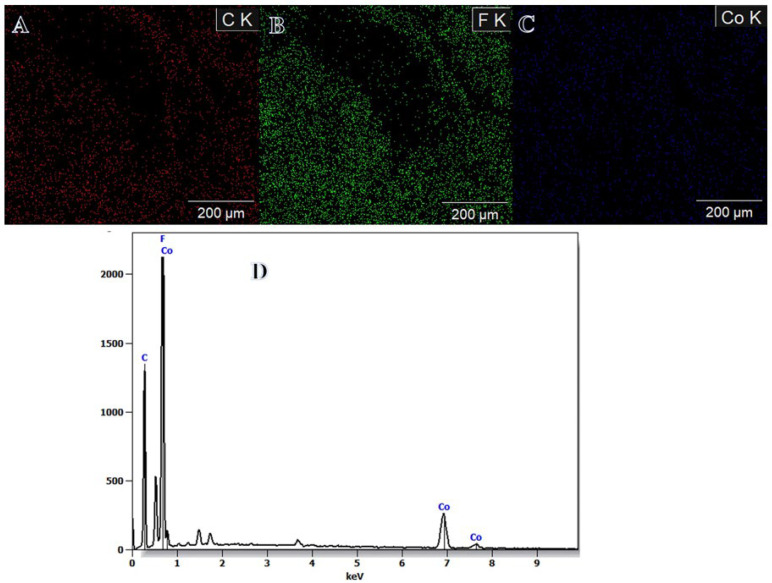
Mapping distribution of carbon (**A**); fluorine (**B**); cobalt (**C**) in the PVHF-40 membrane, and (**D**) EDX result of mapping image.

Dehydrogenating NaBH_4_ using methanol rather than water offers certain benefits. There are a few different explanations, one of which has to do with the by-product itself. NaB(OCH_3_)_4_ is formed during methanolysis; in contrast to NaB(OH)_4_, it does not have a high tendency to polymerize into polyborates. This aids in preventing their precipitation, which may lead to clogged pores and poisoned catalysts [55,56]. Hongming et al. [3] used hydrothermal and reduction approaches to produce Co NPs@carbon nanostructures for SBH dehydrogenation. As sodium metaborate is insoluble in ethanol, they reported that ethanol solution media reduced Co ions better than water media, where some sodium metaborate precipitated with the Co NPs. This helped to separate and avoid the agglomeration of the Co nanoparticles. Once and for all, DI water was used to remove the sodium metaborate. Surfaces of the electrospun Co^2+^/PVFH membranes had a deep purple hue. The formation of Co NPs on the surface of PVFH membranes is shown by a change in color to a dark brown upon contact with the reducing solution, indicating the chemical reduction of Co ions. In other words, the PVFH membranes’ surfaces might be completely covered in an epidermal layer of black Co dots, similar to shell “Co nanoparticles”-core “polymeric NFs” arrangements. On the PVFH-40 membrane, an elemental map is depicted as shown in Figure 2A–C. The SEM pictures clearly show that Co NPs are abundantly distributed throughout the NFs. Mapping EDX demonstrated the presence of carbon, cobalt, and fluorine peaks.

**Figure 3:**
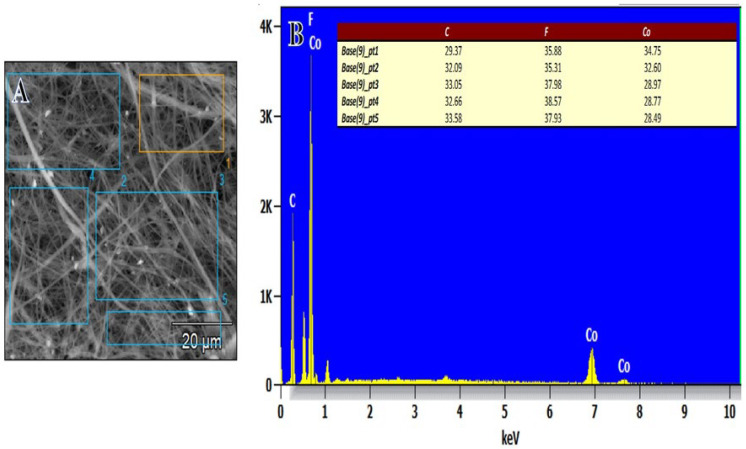
(**A**) SEM picture and (**B**) EDX graph of PVHF-40 membrane at different SEM image points.

Figure 3A,B SEM-EDX shows a graph of the PVHF-40 membrane. The production of Co NPS around the PVDF-HFB nanofibers is confirmed by inspection of many points of the SEM image. It was confirmed that these composite membranes were successfully made thanks to the detection of carbon, cobalt, and fluorine peaks that are all relevant to their production. It is also confirmed that the amounts of produced elements are agreement with the used precursors.

**Figure 4:**
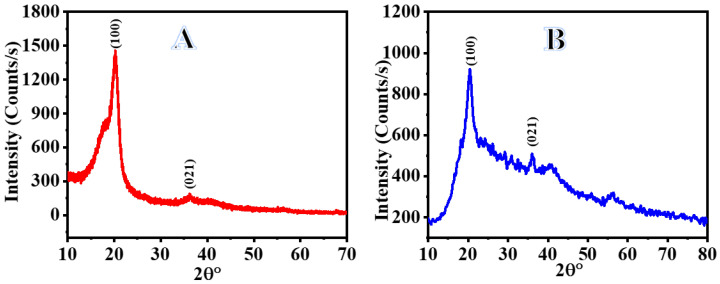
XRD charts of PVHF (**A**) and PVHF-40 (**B**) membranes.

Figure 4 shows the results of an XRD analysis of PVHF-40 membrane crystallinity. In the XRD graph of the PVHF membrane, the three main diffraction planes were found at 2 theta of 20.49°and 36.18°, which correspond to the (100) and (021) planes, respectively [57]. On the other hand, XRD does not reveal any Co NPs in the sample, perhaps because the cobalt NPs are too tiny or amorphous [22,58,59].

**Figure 5:**
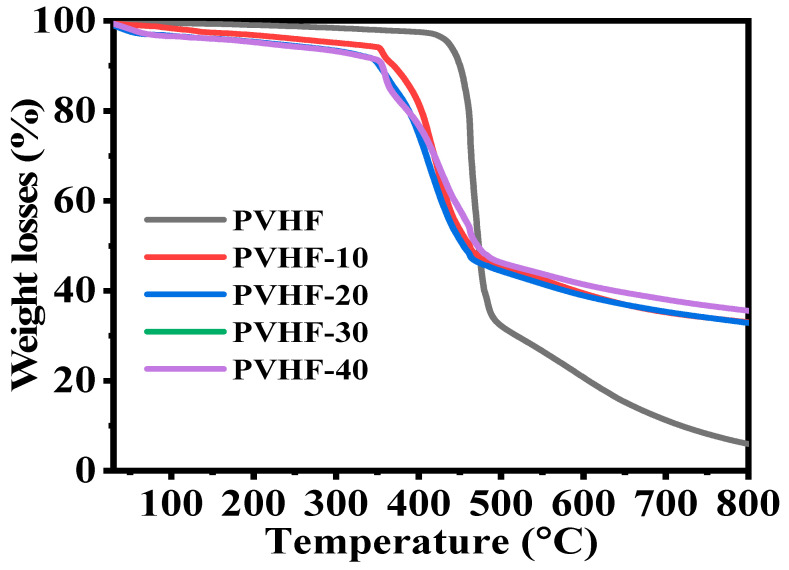
TGA graph of PVHF and Co@PVHF membranes.

TGA charts (Figure 5) were used to investigate the thermal decomposition of PVHF and Co@PVHF membrane NFs. Bare PVHF film had a distinct weight loss region at 420 °C as a result of the stochastic bond cleavage of its units during decomposition [60,61]. After cobalt species were introduced into the PVHF film, this transition was seen at 346 °C, in addition to a little shift observed at 95 °C, which could be due to the evaporation of physisorbed water. The van der Waals forces between the chains of this polymeric membrane were reduced due to the incorporation of cobalt nanoparticles into its structure. As a result, metal-supported polymeric membranes degraded at lower temperatures compared to naked mat [62].

**Figure 6:**
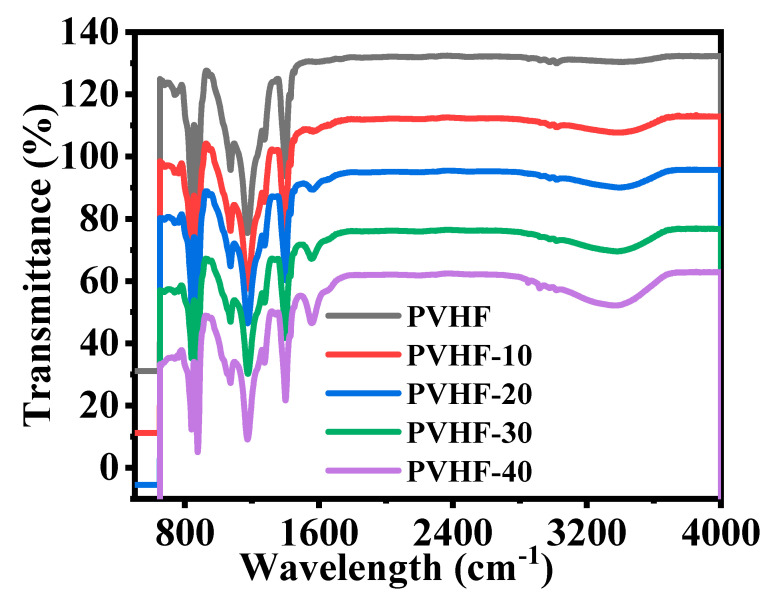
FTIR graphs of PVHF and Co@PVHF membranes.

Figure 6 shows the FTIR of PVHF and Co@PVHF membranes. Both of the plots representing the polymeric membrane had common vibrational bands and phases that were validated by peaks at 749 and 837 cm^−1^ [63]. In the non-crystalline phase of PVHF membrane, there were two additional vibrational bands, at 672 and 872 cm^−1^, which were attributed to CF and CH_2_ movement of vinylidene units, respectively. Bands at 1071, 1175, and 1400 cm^−1^ [23] also demonstrated the symmetric CF stretching, CF_2_ stretching, and deformed vibrations in this membrane. With the addition of cobalt precursor salt during the production of the polymeric film, two new peaks formed. In this nanomaterial, the stretching vibration maxima at 942 and 1561 cm^−1^ [64] provided more evidence for the incorporation of CoO species.

### Dehydrogenation of SBH

**Figure 7:**
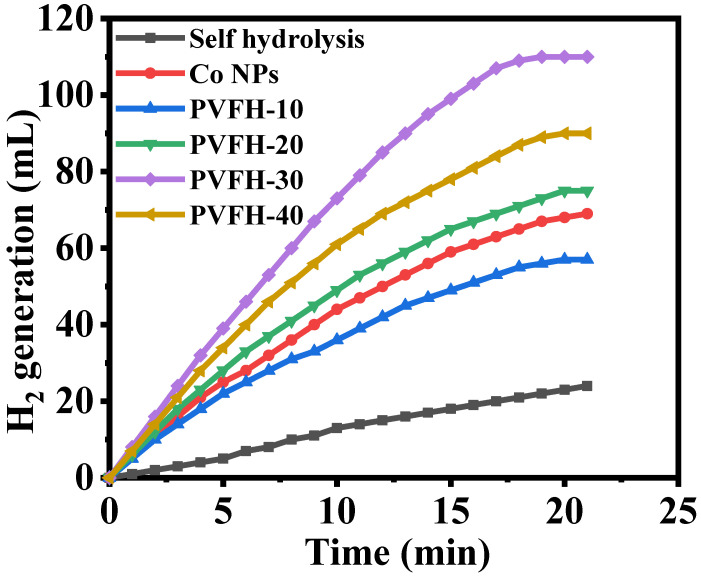
The influence of Co@PVHF membranes on the dehydrogenation of SBH. The amount of catalyst = 100 mg, [SBH] = 1 mmol, and T = 298 K.

The catalysts’ catalytic activity varied widely depending on their shape. To improve catalytic performance, shape-anisotropic nanostructures were used, which included a greater number of active catalytic sites. Nanofibers may be superior to other nanostructures as catalytic supports because of their larger surface area. The long axial ratio associated with nanofibrous morphologies, however, has been proven to result in a significant performance boost over other nanostructures. In an electrospinning-based synthesis, Co@PVHF membranes were investigated as a catalyst for the hydrolysis of SBH, where they were shown to be very active in the generation of H_2_. SBH self-hydrolysis produces H_2_ that is almost equal to that produced in the presence of pure PVHF. Furthermore, it demonstrated a much slower process than H_2_ production in the presence of a Co@PVHF membrane (Figure 7). Kinetic barriers that inhibit significant increases in H_2_ yield from SBH hydrolysis in the absence of a catalyst and at ambient conditions when pure PVHF is present. It was also observed that the naked Co NPs demonstrated high catalytic activity compared to PVHF-10. However, to separate and reuse the catalyst, membrane NFs excelled above traditional powder-like catalysts.

As shown in Figure 7, the impact of Co loading was studied. To find out which formulation of the synthesized MNFs was the most active, 100 mg of MNFs was added to 1 mmol of SBH at 25 °C in the sealed glass reactor. For four different catalyst loadings, the amount of H_2_ produced significantly differed from that with MNFs-free Co. This result indicates that the process can be catalyzed effectively with a high catalyst loading. Insights reveal PVHF-40 to be a potent catalyst for the hydrolysis of SBH, leading to the release of H_2_ (Figure 7). In comparison to the other ratios (PVHF-10 (56 mL), PVHF-20 (73 mL), and PVHF-20 (89 mL)), in 19 min the PVHF-40 (110 mL) MNFs generated the most H_2_, hence they were selected for further testing. In addition, the reaction time for hydrolysis was sped up when Co loading is raised, and more hydrogen H_2_ was produced. More highly efficient catalytic active sites are the cause of this phenomenon. Synthesized MNFs outperformed Ni-PVF [65], PVF-[C6(mpy)_2_][NiCl_4_]_2_ NFs composite [25], and PVF/CoCl_2_/Y-Zeolite [35].

**Figure 8:**
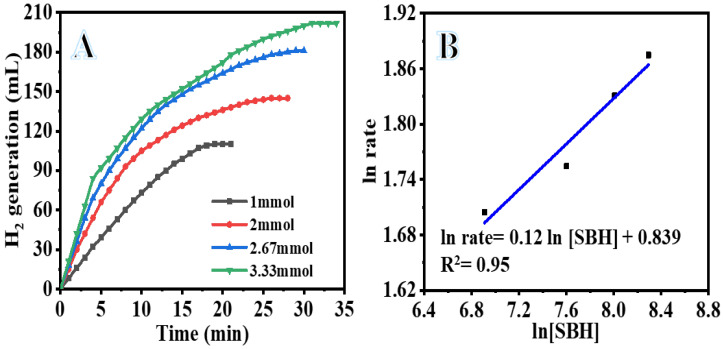
The influence of SBH concentration on the dehydrogenation process (**A**) and ln H_2_ generation rate vs. ln [SBH] (**B**). The amount of catalyst = 100 mg and T = 298 K.

Factors such as SBH concentration, reaction temperature, catalyst amount, and catalyst reusability all have an impact on the efficiency of catalytic hydrolysis of SBH. As a direct consequence of this, investigation into the influence of these factors on SBH hydrolysis while PVHF-40 was present was carried out. The concentration of SBH is essential to the kinetics of the catalytic reaction since it is the source of hydrogen that is produced during the hydrolysis process. The influence of SBH concentration on the rate of H_2_ production was investigated at a temperature of 25 °C using 100 mg of PVHF-40. The catalytic activity of PVHF-40 is shown in Figure 8A. This activity was performed in the presence of varying doses of SBH. As demonstrated in the figure, the H_2_ production rose considerably as the SBH concentration was increased from 1 mmol to 3.3 mmol. This is because a low concentration of SBH was used, since greater concentrations follow the zero order reaction, as the viscosity rises with increasing SBH concentrations. As a consequence of the development of a low solubility by-product (NaBO_2_) during SBH hydrolysis, which is capable of adsorbing to the surface of the catalyst, the reactant diffusion resistance and reaction rate decrease, which ultimately leads to the blocking of active sites [66,67,68,69]. Comparatively to the studies that reveal a zero-order response, our research was conducted at a low concentration. The relationship between the H_2_ production rate and the SBH concentration is seen in Figure 8B. The H_2_ generation rate adheres to pseudo-zero order kinetics (slope = 0.123) in terms of the SBH concentration.

**Figure 9:**
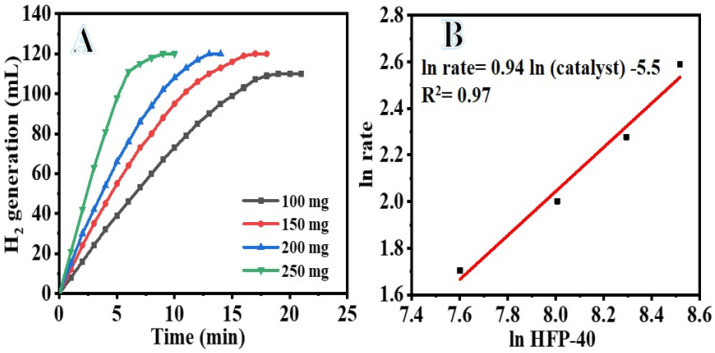
The influence of PVHF-40 membrane amount on dehydrogenation of SBH (**A**) and the ln H_2_ generation rate vs. ln PVHF-40 amount (**B**). [SBH] = 1 mmol and T = 298 K.

Changing the concentration of the catalyst used in the hydrolysis process is a simple and effective way to regulate the rate at which H_2_ is produced (Figure 9). Since additional catalyst gives more active sites for SBH dehydrogenation, increasing the amount of PVHF-40 significantly enhanced the cumulative volume of H_2_ produced during SBH hydrolysis. H_2_ is produced by the self-SBH hydrolysis process, although it moves extremely slowly and eventually ceases (Figure 6). Since the SBH hydrolysis process is a catalyst-controlled reaction, effective H_2_ production may be attained in the presence of a suitable amount of catalyst (Figure 9A). As the amount of catalyst is increased, the volume at which H_2_ is produced increases, as depicted in the figure. The H_2_ generation volume increased from 110 mL in 19 min to 120 mL in 9 min after increasing the amount of catalyst from 100 to 250 mg. A phenomenon known as “structure sensitivity” explains this observation; as the number of active sites in a catalyst rises, the reaction rate also increases. Thus, it was evident that the amount of catalyst used governs the rate at which H_2_ is produced. The rate of ln H_2_ production vs ln [PVHF-40] is shown in Figure 9B. The best-fit line had a slope of 0.95, thus we can infer that the hydrolysis process had pseudo-first order kinetics with respect to the concentration of the catalyst.

**Figure 10:**
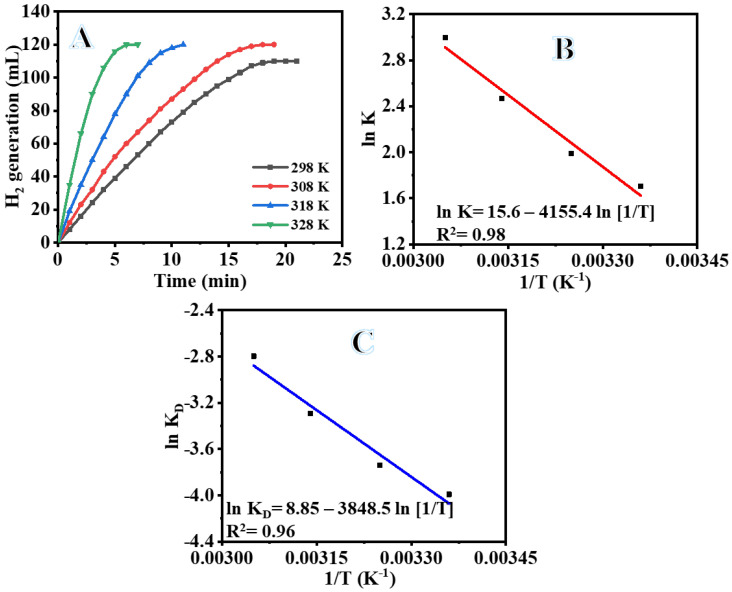
The influence of temperature on dehydrogenation of SBH (**A**), ln K vs. (1/T) (**B**), and ln K_D_ vs. (1/T) (**C**). The amount of catalyst = 100 mg and [SBH] = 1 mmol.

As was to be anticipated, raising the reaction temperature led to a rise in the volume production of H_2_ and resulted in a linear relationship between H_2_ volume and reaction time (Figure 10A). Because of the higher temperature, the time required for the reaction that results in the production of H_2_ decreased, as can be seen in the figure. In addition, the volume of H_2_ produced was raised. As may be seen in Figure 10, an increase in the reaction temperature from 25 to 55 °C caused an increase in the H_2_ production as 100% H_2_ was release in 6 min. The findings of this study are in agreement with those in previous research [70,71,72]. The activation energy (E_a_) of dehydrogenation of SBH hydrolysis using the Arrhenius Equation (4) in the temperature range from 25 to 55 °C in the presence of 100 mg PVHF-40 and 1 mmol SBH is as follows:

(4) $$lnk=lnA-\frac{E_{a}}{RT}$$

**Figure 11:**
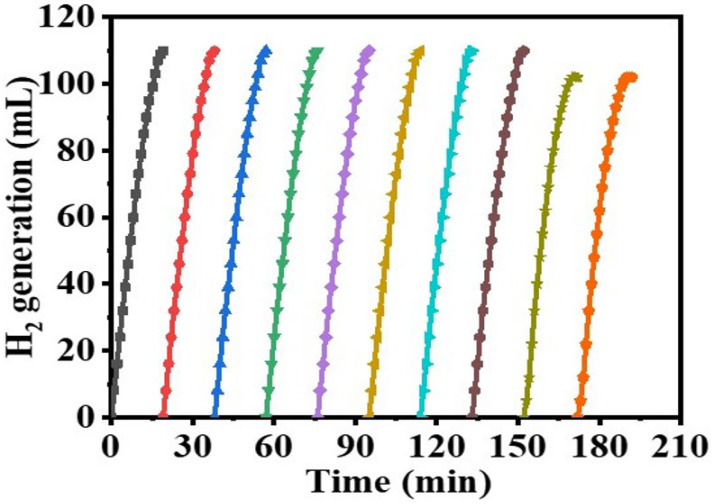
Reusability test of PVHF-40. The amount of catalyst = 100 mg, [SBH] = 1 mmol, and T = 298 K.

The linear portion of temperature graphs is used to calculate the rate (k). Figure 10B shows an Arrhenius plot of ln (K) versus 1/T. As seen in Figure 10B and the Arrhenius equation (Equation (4)), Ea = 35.21 kJ mol-1 was found. The literature reports activation energies for non-noble metals ranging from 16.28 to 42.45 kJ mol^−1^ [73,74,75,76,77]. The obtained E_a_ value was compared with the E_a_ values of various Co-based catalysts and catalysts supported on polymer substrate. Some examples of these catalysts that can be found in the literature include Co-Ni/AC (68.84 kJ mol^−1^) [78], Ni- PVF capsules (49.3 kJ mol^−1^) [53], Ni-PVF hollow fiber (55.3 kJ mol^−1^) [41], ([C6(mpy)_2_][NiCl_4_]^2−^ (56.36 kJ mol^−1^) [79], PVF-[C6(mpy)_2_][NiCl_4_]^2−^(44.54 kJ mol^−1^) [25], Co-Ni/MWAC (40.7 kJ mol^−1^) [76], Sm-Ni-Co-P/g-Al_2_O_3_ (52.05 kJ mol^−1^) [72], Ni–Co–B (62 kJ mol^−1^) [80], indicating a superior catalytic performance of the introduced Co@PVHF. The catalyst’s suitability for practical use is largely dependent on its reusability and stability. After the reaction, the Co@ PVHF catalysts may be separated simply by withdrawing the MNFs from the catalytic system. Separation as easy as that has a lot going for it. Without separating or activating the catalyst, SBH may be refilled into the reaction solution (1 mmol of SBH at 25 °C) (100 mg PVHF-40). For this reason, several cycles of SBH hydrolysis were performed to look into PVHF-40s for recycling. As the number of cycles rises, slight declines are seen (Figure 11). However, the same volume of H_2_ was generated by extending the reaction time. This may be because, when the membrane is reused without cleaning among cycles, reaction products precipitate out onto its surface, blocking the metal active sites and slowing down hydrogen production.

**Figure 12:**
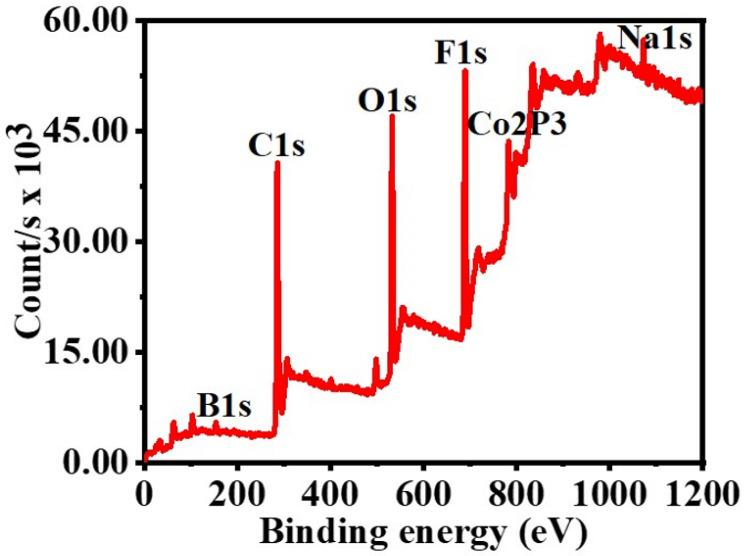
XPS spectrum of PVHF-40 membrane after ten cycles reused.

It has been hypothesized that a rise of NaBO_2_ precipitation on the PVHF-40 surface and the consequent rise in viscosity of the solution [81], both of which impede active site accessibility or induce pore blockage, are responsible for the modest drop in the catalytic activity seen after the fifth cycle. This was confirmed by XPS of the PVHF-40 catalyst after ten cycles (Figure 12). As seen by the Co2p3 peak at 857 eV, Co(OH)_2_ was formed after six cycles of reuse. A Na 1s peak (1072.2 eV) and a B 1 s peak (180 eV) both showed up in the spectra. One possible explanation is that Na ions, which are predominantly impurities that are dissolved in the solution during dehydrogenation of SBH, are responsible for this. The results indicate that Co@PVHF performs well as a catalyst for SBH hydrolysis.

The kinetics equation of the dehydrogenation processes could be expressed as Equations (5)–(7).

(5) $$r=-\frac{d\left[SBH\right]}{dt}=k{\left[PVHF-40\right]}^{0.123}{\left[SBH\right]}^{0.95}$$

(6) $$\begin{array}{c} k=Ae^{\left(\frac{-E_{a}}{RT}\right)}\to lnk=ln15.84-\frac{35210}{8.314T} \end{array}$$

(7) $$\begin{array}{c} r=-\frac{d\left[SBH\right]}{dt}=15.84e^{\left(\frac{-4235}{T}\right)}{\left[PVHF-40\right]}^{0.123}{\left[SBH\right]}^{0.95} \end{array}$$

(8) $$\begin{array}{c} lnk_{D}=ln\frac{k_{B}}{h}+\frac{\Delta S}{R}-\frac{\Delta H}{RT} \end{array}$$

(9) $$\begin{array}{c} \Delta G=\Delta H-T\Delta S \end{array}$$

Based on Equation (9) and Figure 10C, ΔH and ΔS were determined to be 32.61 kJ mol^−1^ and 0.075 kJ mol^−1^, respectively. ΔG can be rewritten as follows:

(10) ΔG=32.61−0.075T

## 4. Conclusions

In conclusion, the electrospinning method, followed by in situ reduction of Co ions to metallic Co, successfully produced the Metallic Co NPs@PVHF membrane NFs catalysts. Excellent catalytic activity in the dehydrogenation of SBH was observed with the synthesized metal catalysts. When compared to the other formulations, the sample PVHF-40 had the highest catalytic activity. In terms of SBH concentration and catalyst concentration, the kinetics investigation revealed that the reaction followed the pseudo-zero order and pseudo-first order, respectively. The rise in temperature from 25 to 55 °C resulted in an increase in H_2_ generation yield to be 100% in 6 min and a low activation energy (35.21 kJ mol^−1^) was obtained. More intriguingly, the Co NPs@PVHF MNFs catalyst was utilized without separation or makeup across six consecutive cycles of the reactions. Given that the eco-friendly and low-cost Co@PVHF MNFs are catalytically effective and reusable, they should have potential applications in the dehydrogenation of SBH. The findings might pave the way for additional research into effective and practical catalysts for on-demand H_2_ generation via SBH hydrolysis.

## Data Availability

The data presented in this study are available from the corresponding authors upon reasonable request.
